# B Cell Depletion in HIV-1 Subtype A Infected Ugandan Adults: Relationship to CD4 T Cell Count, Viral Load and Humoral Immune Responses

**DOI:** 10.1371/journal.pone.0022653

**Published:** 2011-08-23

**Authors:** Peter Oballah, Britta Flach, Leigh A. Eller, Michael A. Eller, Benson Ouma, Mark de Souza, Hannah N. Kibuuka, Fred Wabwire-Mangen, Bruce K. Brown, Nelson L. Michael, Merlin L. Robb, David Montefiori, Victoria R. Polonis

**Affiliations:** 1 Makerere University Walter Reed Project, Kampala, Uganda; 2 Military HIV Research Program, Rockville, Maryland, United States of America; 3 The Henry M Jackson Foundation, Rockville, Maryland, United States of America; 4 Armed Forces Research Institute of Medical Sciences, Bangkok, Thailand; 5 Makerere University School of Public Health, Kampala, Uganda; 6 Walter Reed Army Institute of Research, Rockville, Maryland, United States of America; 7 Duke University, Durham, North Carolina, United States of America; University of Cape Town, South Africa

## Abstract

To better understand the nature of B cell dysfunctions in subjects infected with HIV-1 subtype A, a rural cohort of 50 treatment-naïve Ugandan patients chronically infected with HIV-1 subtype A was studied, and the relationship between B cell depletion and HIV disease was assessed. B cell absolute counts were found to be significantly lower in HIV-1+ patients, when compared to community matched negative controls (p<0.0001). HIV-1-infected patients displayed variable functional and binding antibody titers that showed no correlation with viral load or CD4+ T cell count. However, B cell absolute counts were found to correlate inversely with neutralizing antibody (NAb) titers against subtype A (p = 0.05) and subtype CRF02_AG (p = 0.02) viruses. A positive correlation was observed between subtype A gp120 binding antibody titers and NAb breadth (p = 0.02) and mean titer against the 10 viruses (p = 0.0002). In addition, HIV-1 subtype A sera showed preferential neutralization of the 5 subtype A or CRF02_AG pseudoviruses, as compared with 5 pseudoviruses from subtypes B, C or D (p<0.001). These data demonstrate that in patients with chronic HIV-1 subtype A infection, significant B cell depletion can be observed, the degree of which does not appear to be associated with a decrease in functional antibodies. These findings also highlight the potential importance of subtype in the specificity of cross-clade neutralization in HIV-1 infection.

## Introduction

Human immunodeficiency virus (HIV) infection leads to dysregulation of the host immune system resulting in acquired immunodeficiency syndrome (AIDS), opportunistic infections, malignancies and eventual death. In the majority of untreated cases, infection with HIV-1 ultimately results in elevated viral replication leading to depletion and impairment of CD4+ T cells [1], [2] one of the primary markers used for monitoring patients and characterizing disease progression. Chronic HIV-1 infection also leads to B cell dysfunction through mechanisms that are poorly understood [3], [4]. While an intact memory B cell compartment is required to guard against future infections [5], in HIV-1 chronic infection, circulating memory B cells have been observed to be markedly reduced, potentially as a result of increased apoptosis [6], [7]. HIV-1 induces numerous B cell abnormalities, including hypergammaglobulinemia and B cell hyperactivation [8], [9], [10] B cell exhaustion [11], increased expression of activation markers [12], spontaneous secretion of antibodies in culture [13], and a higher incidence of B-cell lymphomas [14]. Persons with chronic HIV-1 infection also show impaired humoral responses to vaccination and their B cells respond poorly to *in vitro* stimulation [15]. Importantly, the early initiation of anti-retroviral therapy soon after HIV infection has recently been shown to preserve the memory B cell compartment and minimize damage to B cell responses in HIV infection [16]. Memory B cells are vital for the maintenance of antibody levels and rapidly initiate secondary immune responses upon re-infection or antigenic stimulation [17]. Antigen-induced B cell proliferation and differentiation is dependent on direct cross-talk with CD4+ T cells, however soluble gp120 can interfere with this interaction [18]. If this interaction is disrupted, germinal center reactions are inhibited, the microenvironment for somatic hypermutation will not be established and thus, B cell differentiation may be aborted. In HIV-1 infection, elevated viral plasma load and disease progression have also been shown to be associated with loss of B cell reactivity [19].

More than 33 million people are infected with HIV-1 worldwide and a preventive vaccine is urgently needed. It has been proposed that an efficacious HIV vaccine will require effective T cell immunity, as well as cross-reactive, functional antibodies. Neutralizing antibody (NAb) responses to HIV-1 are therefore a high priority for HIV-1 vaccine development [20], [21]. Cross- subtype NAbs have been found in the sera of HIV-1 infected individuals and numerous studies have reported preferential recognition and inhibition of preceding autologous viral strains, implying that HIV-1 quickly escapes selective antibody pressure [22], [23], [24], [25]. Nevertheless, some patients do demonstrate potent, broadly cross-reactive neutralization by targeting epitopes of the HIV-1 envelope protein. The relationship between these responses and disease progression in subjects infected with HIV-1 subtypes other than B has been characterized in a limited number of studies [26], [27], [28].

Another functional HIV-1 antibody response, antibody-dependent cell-mediated cytotoxicity (ADCC), has been correlated with viral load and rate of progression to AIDS [29], [30], [31], [32], [33]. Despite substantial research to reveal the presence and magnitude of ADCC at different stages of HIV-1 disease [29], [30], [34], [35] and the potential protective effect of this response in vaccinated animal models [36], [37], [38], the relevance of ADCC in HIV-1 infection is still unclear. HIV-1 specific immune responses such as ADCC and cytotoxic CD8+ T-cells are likely to lead to destruction of HIV-1-infected CD4+ T cells resulting in gradual loss of T cell “help” to B cells, thereby contributing to a reduction in B cell numbers and dysfunctions in antibody secretion. In contrast to HIV-1-specific NAbs that are known to deter free virus by blocking receptor engagement, interfering with the fusion process, or by other mechanisms [39], the roles of many other binding antibodies elicited in natural infection are poorly understood. Additional functional consequences of binding antibodies may include pathogen opsonisation, inhibition of bystander cell apoptosis, or enhancement of infection.

Given the limited number of studies performed to explore B cells, antibody function and markers of disease progression in non-B subtype infections, we have characterized the peripheral blood B cell absolute counts and serum antibody functions in untreated HIV-1 subtype A infected Ugandan patients. These subjects were enrolled in a vaccine cohort development protocol in Kayunga district in rural Uganda where the HIV-1 prevalence was 9.9% and the subtype distribution was found to be 45% subtype A, 23% D, 1% C, 23% recombinants and 8% represented dually reactive subjects [40]. Here we show the impact of HIV-1 subtype A-associated B cell depletion on overall binding antibodies, NAb responses and ADCC activity. We demonstrate for the first time an inverse correlation between B cell number and NAb titers against a panel of ten acute pseudoviruses from five different subtypes. Additionally, the NAbs from subtype A-infected subjects demonstrate subtype preference with more frequent responses and a higher geometric mean titer against subtype A-containing pseudoviruses. These findings may provide important information for vaccine development in East Africa, where HIV-1 subtypes A, C and D are the most prevalent [41].

## Materials and Methods

### Subjects

Study participants were recruited from a cohort established for possible HIV-1 vaccine trials, as previously reported [40]. Samples were obtained from 192 HIV-1 infected, treatment naïve subjects and 262 HIV-seronegative individuals; 50 subtype A HIV-1 infected subjects were randomly selected for study in antibody assays. This study was approved by institutional review boards in the US, Division of Human Subjects Protection at the Walter Reed Army Institute of Research and the institutional Review Boards of Uganda's National Council for Science and Technology (UNCST) and the National AIDS Research Committee (NARC). Study participants were aged 15–49 years and provided written informed consent before blood draw. Written assent was obtained from the parent or legal guardian of those participants aged 15–17.

### HIV-1 testing, viral load determination, and immunophenotyping

Sera were screened for HIV-1 by ELISA using the Genetic Systems rLAV ELISA (BioRad Laboratories, Redmond, WA), followed by testing of reactive samples in duplicate using the Vironostika HIV-1 Microelisa Systems (Organon Teknika, Durham, NC). Samples repeatedly reactive were confirmed by Genetic Systems HIV-1 Western Blot (BioRad Laboratories, Redmond, WA). Viral burden was measured on plasma using the HIV-1 Monitor Test version 1.5 (Roche Diagnostics, Indianapolis, IN). Virus subtype analysis was performed on plasma using the multi-region hybridization (MHA) assay for subtypes A, C, and D that was designed to identify HIV-1 subtypes, recombinants, and dual infections in a rapid, high throughput, real-time PCR (Applied Biosystems 7900HT Fast Real-Time PCR, Applied Biosystems, Carlsbad, CA) [42], [43]. Enumeration of lymphocyte subsets was performed using FACS MultiTEST System that utilizes Multi-test 4-color reagents and TruCount tubes in a single platform flow cytometry based system (Becton Dickson (BD), San Jose, CA). Samples were aquired and analyzed on a dual laser BD FACSCalibur. B cell subsets were defined using the anti-human CD19, clone SJ25C1 conjugated to allophycocyanin (APC).

### TZM-bl cells

TZM-bl cells were obtained from NIH AIDS Reagent program, as contributed by John Kappes and Xiaoyun Wu. TZM-bl are genetically engineered cells, derived from a HeLa cell line. They express CD4, CXCR4 as well as CCR5 and contain Tat-responsive reporter genes for firefly luciferase and *Escherichia coli* β-galactosidase under regulatory control of an HIV-1 long terminal repeat (LTR) [44], [45]. The cells were maintained in Dulbecco's modified Eagle's medium (Invitrogen, Carlsbad, CA) containing 10% heat-inactivated fetal bovine serum and gentamicin (50 µg/ml). Cells were incubated at 37°C with 5% CO_2_. Cell monolayers were split 1∶10 at confluence by treatment with 0.25% trypsin, 1 mM EDTA (Invitrogen).

### Pseudoviruses

Molecularly cloned gp160 genes representing standard panels of HIV-1 reference strains for subtypes A (92RW020.2, Q842.d12, Q23.17), B (WITO4160.33, QH0692.42), C (Du172.17, CAP45.2.00.G3), D (A08483M1.vrc9a), and CRF02_AG (PT271.11, PT250.4), from newly transmitted, sexually acquired infections were used in the neutralization assays. All env-pseudotyped viruses were prepared by co-transfection with an env-defective backbone plasmid (pSG3Δenv) in 293T cells and titrated in TZM-bl cells.

### Neutralizing antibody assay

Neutralizing antibodies were measured as a function of reduction in luciferase reporter gene expression after a single round of infection in TZM-bl cells, as described [46]. Briefly, virus was pretitered to determine the dilution of pseudovirus sufficient to produce 150,000 relative light units (RLU) from luciferase expression at day 2 post-infection. This dilution of virus then was incubated with serial 3-fold dilutions of heat-inactivated sera in duplicate in a total volume of 150 µl for 1 hr at 37°C in 96-well flat-bottom culture plates. Freshly trypsinized cells (10,000 cells in 100 µl of growth media containing 25 µg/ml DEAE dextran) were added to each well. One set of control wells received cells plus pseudovirus (virus control) and another set received cells only (background control). After a 48 hr incubation, 150 µl of culture medium was removed from each well and 100 µl of Britelite reagent (PerkinElmer, Waltham, MA) was added to the cells. After a 2-minute incubation at room temperature to allow cell lysis, 150 µl of cell lysate was transferred to Costar® 96-well black solid plates (Corning, Lowell, MA) for measurements of luminescence using a Victor2™ luminometer (Perkin Elmer). The 50% inhibitory dose (ID_50_) was defined as the serum dilution that caused a 50% reduction in RLU compared to virus control wells, after subtraction of background RLU.

### Binding antibody assay

IgG antibody titers were measured in plasma using recombinant envelope glycoprotein subtype A gp120 (Aalto Bio Reagents Ltd., Dublin, Ireland). 96-Well ELISA plates were coated overnight at 4°C with 100 µl PBS/Well containing anti-gp120 antibody. Plates were washed three times with PBS/0.05% Tween-20 wash buffer. Half the plate was then coated with gp120 antigen (0.5 µg/ml) in distilled water and incubated at 37°C for 1 hr. Plates were washed three times with wash buffer and blocked with diluent containing 5% dry milk for 1 hr at 37°C. Samples were serially diluted and added to the ELISA plate (100 µl/well) for 1 hr at 37°C. After washing five times, a secondary peroxidase-labeled goat anti-human IgG (H+L) (KPL, Gaithersburg, MD), diluted 1∶5000 in wash buffer was added (100 µl/well). Plates were incubated for 1 hr at 37°C, washed three times and developed with TMB substrate (KPL) for 20 minutes at room temperature. The plates were analysed at 405 nm with an EL_X_ 800 universal microplate reader (Bio-Tek Instruments, Winooski, VT). After subtraction of the background, endpoint titers for each sample were established as the last dilution with a corrected optical density of greater than 0.1.

### ADCC assay

The ADCC assay was performed as described previously [47]. Briefly, 2×10^6^ CEM.NK^r^ cells were labelled with 200 µCi radioisotope Na^51^CrO_4_ (^51^Cr) for 1 hr at 37°C. Following washing to remove excess chromium, the cells were incubated with HIV-1 recombinant gp120 subtype B protein (Protein Sciences Corp, Meridian, CT) at 1 µg (10 µl) per 1×10^6^ cells in a total volume of 300 µl and unbound gp120 was washed out. Target cells were plated at 50 µl per well from a target stock of 5×10^4^ cells/ml and incubated for 20 minutes at room temperature (20 to 30°C) with 50 µl of 10-fold serial dilutions (10^2^ to 10^6^) of heat inactivated participant sera. A serum sample with demonstrable ADCC activity was used as positive control and also to normalize the assay. Peripheral blood mononuclear cells (PBMC) derived from a single HIV-1 seronegative Thai donor were used as effector cells. On the day before the assay, cryopreserved PBMC were thawed and incubated overnight and added to the targets at an effector-to-target ratio of 100∶1. The effectors, serum, and target cells were incubated together for 6 hrs. Either sodium dodecyl sulfate (SDS) or cell culture media was added to target cells to measure maximal or spontaneous release, respectively. Chromium release was measured by using a Top-Count NXT™ (Packard Bioscience Company, Meridian, CT, USA). Percentage specific lysis was calculated as 100 [(mean test cpm – mean spon cpm)/(mean max cpm – mean spon cpm)], where test cpm = counts per minute released by the CEM.NK^r^ target cells in the presence of effector cells, spon cpm = cpm released by the CEM.NK^r^ target cells in the absence of any effector cells, and max cpm – cpm released by the target cells in the presence of SDS. In order to normalize results, a positive control was run on every plate on each day the assay was performed and the positive control mean maximum lysis was calculated. Maximum lysis for each sample was calculated as a ratio to the mean maximum lysis of the positive control run on the same day.

### Statistical analyses

Statistics were performed using Prism (GraphPad Software Inc., San Diego, CA) statistical analysis software. Comparisons of groups were performed using the Mann-Whitney test. Correlation was analyzed using the non-parametric two-tailed Spearman's rank test. P values less than 0.05 were considered statistically significant.

## Results

### Absolute B cell counts are significantly lower in HIV-1 subtype A-infected Ugandans, as compared to HIV-1 negative controls

**Figure 1:**
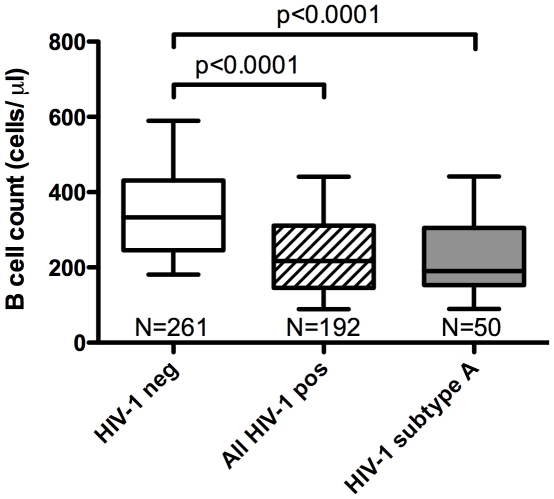
The absolute B lymphocyte count in HIV-1 subtype A infected patients is significantly lower than in HIV-1 negative controls. Samples of whole blood from HIV-1 infected and uninfected individuals were analysed using flow cytometry and the MultiTEST™ IMK Kit (BD). Lymphocyte subpopulations were quantified and samples from 261 HIV-1 negative individuals (white box) were compared with HIV positives. The absolute counts of CD19+ B lymphocytes in 50 HIV-1 subtype A infected patients (grey box) and in 192 patients with all HIV-1 subtypes combined (hatched box) were significantly reduced in comparison to uninfected participants. (p<0.0001, Mann-Whitney test). The whiskers represent the range from the 10th–90th percentile.

The whole blood absolute counts and percentages of T and B cells were measured in 192 treatment-naive patient samples and 261 samples from HIV-1 negative, community matched control subjects. Fifty HIV-1 subtype A infected patients were selected for further humoral analyses. The median B cell absolute count in HIV-1 uninfected subjects was 333 cells/µl with a range of 83–1160 cells/µl, while the median absolute B cell count in subtype A infected patients was 190 cells/µl, with a range of 20–572 cells/µl. Compared to their HIV-1 negative counterparts, all HIV-1 infected subjects and the subset of 50 HIV-1 subtype A infected subjects, when analyzed as separate groups, both showed a significant reduction in absolute B cell numbers (p<0.0001, Fig. 1).

### B cell depletion in HIV-1 subtype A infection is accompanied by a reduction in T-Helper cell number and an increase in viral load

**Figure 2:**
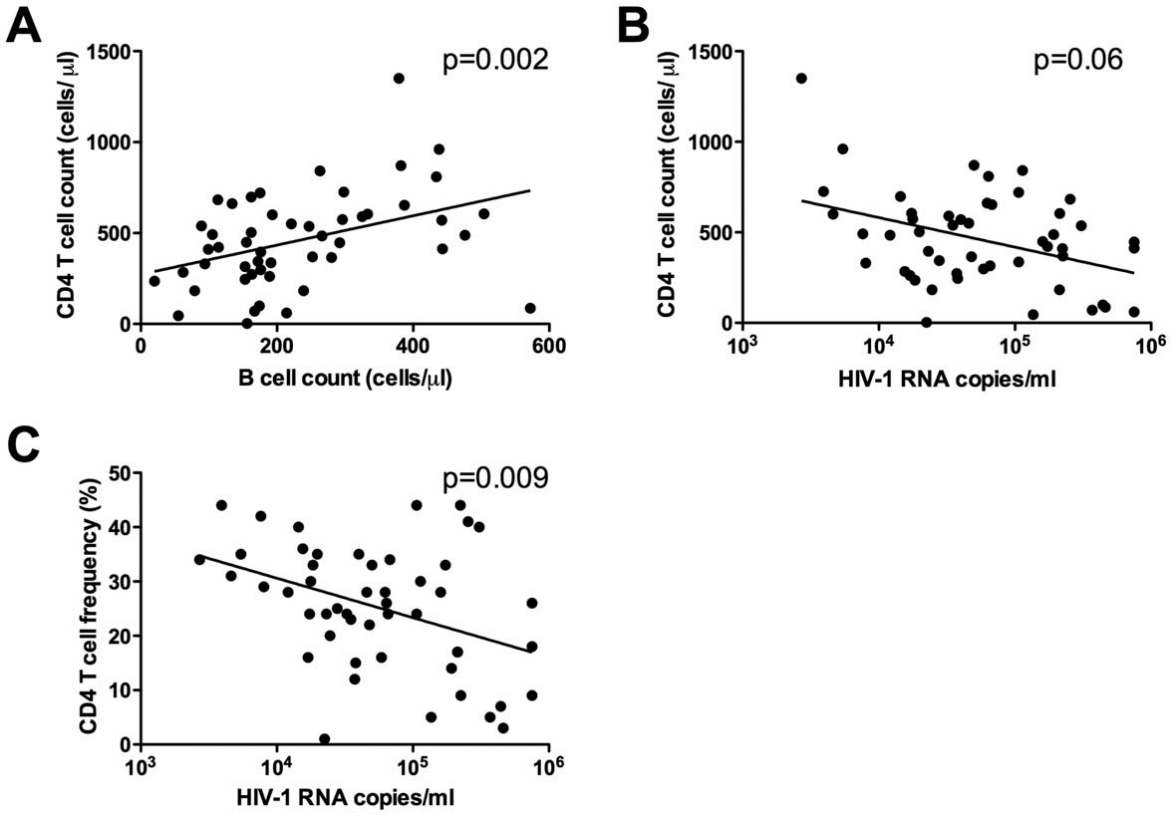
Absolute CD4+ T lymphocyte counts correlate with B lymphocyte absolute counts, but not with HIV-1 viral Load. The absolute counts of B lymphocytes correlated with the absolute number of CD4+ T lymphocytes (A). The number of plasma HIV-1 RNA copies/ml showed a significant inverse correlation with the CD4+ T lymphocyte percentage (C) and a trend towards correlation with CD4 absolute number (B).

To investigate if the observed B cell depletion was related to lower numbers of CD4+ T cells or higher viral loads, the absolute counts of T-helper cells were compared with B cell counts and viral load. As shown in Fig. 2A, the absolute number of B cells correlated directly with the CD4+ T cell count, a primary marker of disease progression in HIV-1 infection (Spearman, p = 0.002, rho = 0.43). The median CD4+ T cell count was 448 cells/µl with a range of 3–1350 cells/µl. The mean viral load (copies of HIV-1 RNA/ml) was 48,989, with a range of 2,702–750,000 copies per ml. Viral load showed an inverse relationship with CD4+ T cells, using both absolute counts (Spearman, p = 0.06, rho = −0.37, Fig. 2B) and percentage (Spearman, p = 0.009, rho = −0.26, Fig. 2C). There was no correlation between viral load and absolute B cell counts (Spearman, p = 0.68, r = −0.06, data not shown).

### B cell numbers in HIV-1 subtype A infected individuals correlate inversely with neutralization titers against different HIV-1 subtypes in the TZM-bl pseudovirus assay

**Figure 3:**
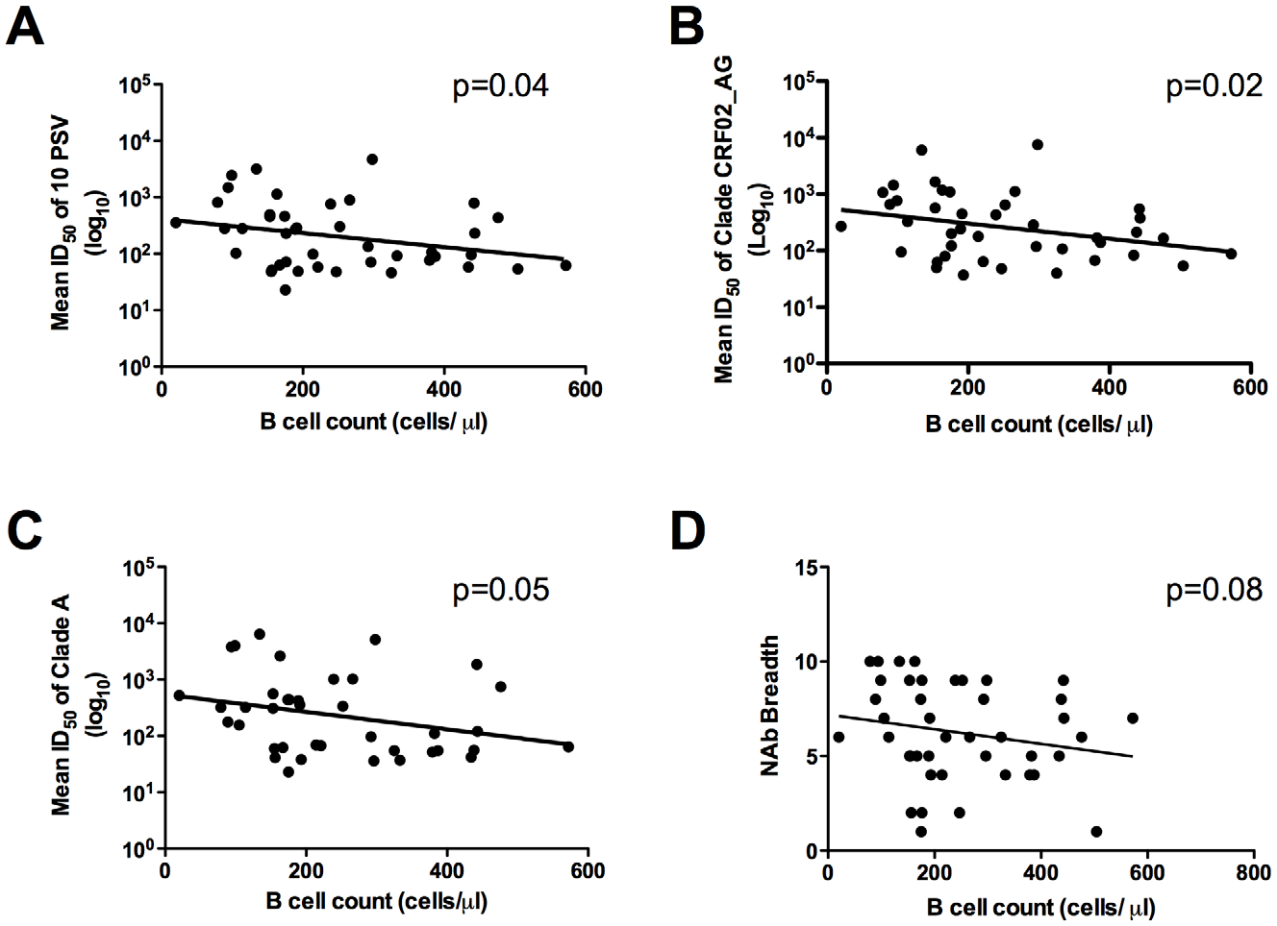
B lymphocyte absolute counts in HIV-1 subtype A infected Ugandans correlate inversely with neutralizing antibody titers against different HIV-1 subtypes. Neutralizing antibody responses in sera of 50 HIV-1 subtype A infected patients were determined against a panel of 10 pseudoviruses in the TZM-bl Assay. The mean ID_50_ using each serum sample against all 10 pseudoviruses or against all pseudoviruses within a subtype was calculated. B lymphocyte numbers showed a significant inverse correlation with neutralizing antibody titers against the mean ID_50_ of all 10 pseudoviruses (A) and with the mean ID_50_ for subtype CRF02_AG (B); the same trend was observed for subtype A viruses (C). B lymphocyte numbers also showed a trend towards inverse correlation with breadth (number of panel viruses neutralized) (D).

To assess the relationship between B cell numbers and functional antibodies, neutralization breadth and potency was assessed using sera from the 50 HIV-1 subtype A infected patients. The TZM-bl neutralization assay was employed using a panel of 10 acute pseudoviruses from 5 different subtypes (see Materials and Methods). The mean 50% neutralizing titers (ID_50_) were calculated for each serum against the pseudoviruses grouped by subtypes (A, B, C, D, CRF02_AG), as well as against all 10 pseudoviruses together. Individual results with an ID_50_≤10 were considered negative and assigned a value of 10, whereas for sera having an ID_50_>10, the actual titer values were included in the analyses. The patients' B cell counts showed a significant negative correlation with the mean ID_50_s against the 10 pseudoviruses (Spearman, p = 0.04, rho = −0.36, Fig. 3A). This was also true for the grouped CRF02_AG pseudoviruses (Spearman, p = 0.02, rho = −0.35, Fig. 3B), and a similar trend was observed for subtype A pseudoviruses (Spearman, p = 0.05, rho = −0.31, Fig. 3C), and for subtype C viruses (p = 0.03, data not shown). A trend was also seen when comparing the B cell absolute counts with the neutralization titers against each of the individual subtype A pseudoviruses, 92RW020.2 and Q842.d12 (Spearman, p = 0.06, p = 0.07, respectively); no additional correlations were observed for the other subtypes or individual viruses (data not shown). The mean ID_50_ values for each of the 50 sera against the 10 different pseudoviruses ranged from 23 to 11,270, with a median of 112. As can be seen in Fig. 3D, there is also a trend towards an inverse correlation (Spearman, p = 0.08) between B cell numbers and the breadth of neutralizing antibody responses (as measured by the total number of panel pseudoviruses neutralized).

### Sera from patients infected with HIV-1 subtype A preferentially neutralize subtype A and the recombinant CRF02_AG

**Figure 4:**
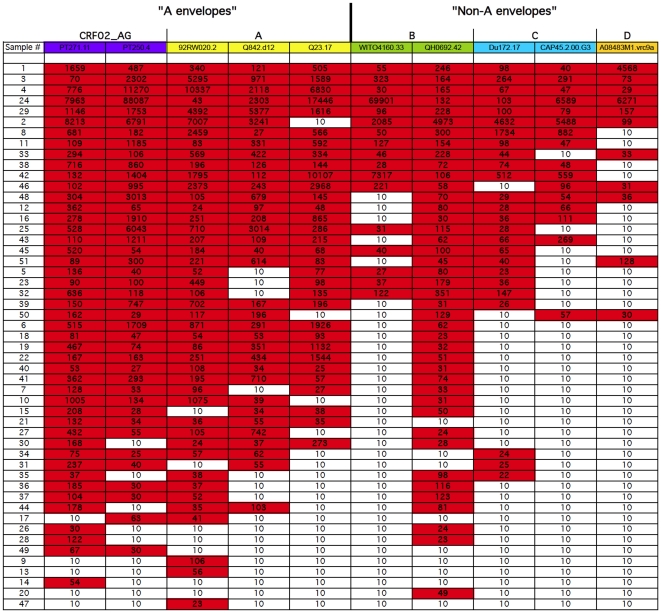
Breadth of neutralization in HIV-1 subtype A infected patient sera. Shown is the breadth of neutralization for each serum sample against a panel of 10 pseudoviruses from five different subtypes (Purple columns = subtype CRF02_AG, yellow = subtype A, green = subtype B, blue = subtype C and orange = subtype D). Red boxes indicate ID_50_s>10 (positive neutralization). HIV-1 subtype A infected subjects showed more frequent neutralization against subtypes CRF02_AG and A, as compared to non-A subtypes (p<0.001, Fisher's exact test).

**Figure 5:**
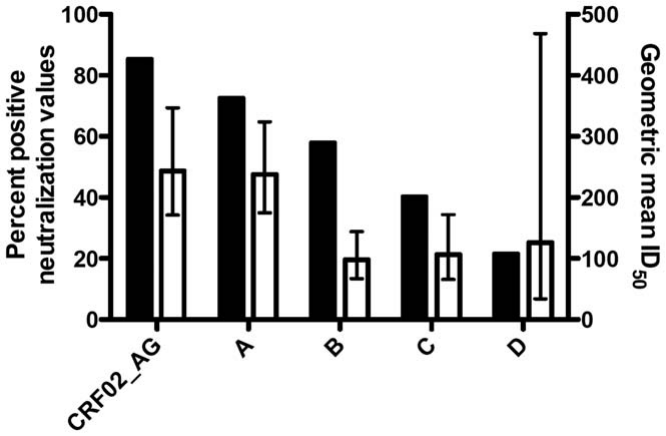
Patients infected with HIV-1 subtype A show greater frequency and magnitude of neutralization against subype A and CRF02_AG viruses. Sera from 50 HIV-1 subtype A-infected patients were tested in the TZM-bl neutralization assay against 1–3 pseudoviruses of each subtype indicated. The frequency of neutralization (% of titers >10) of viruses in a given subtype (black bars), as well as the geometric mean titer (ID_50_, white bars) against each viral subtype, is shown. For the geometric mean titers, the error bars represent the 95% confidence intervals.

To analyze the subtype-specific frequency, breadth and potency of neutralization, the ID_50_ values were tabulated for all 50 subtype A sera against each of the 10 pseudoviruses within the five subtypes, as shown in Fig. 4. Values >10 were highlighted in red boxes, and the sera were ranked from those with highest breadth (top) to those with the lowest (bottom). The breadth of neutralization for each of the different sera ranged from 10% to 100%. Five out of 50 (10%) serum samples could neutralize all ten pseudoviruses (100% breadth), eight sera neutralized nine out of ten pseudoviruses (90% breadth), and only one serum (sample #47) showed a very low breadth of 10% (Fig. 4). For each viral subtype, the number of positive neutralization titers was scored, and the percent of positive neutralization titers was calculated and graphed by subtype, as shown in Fig. 5 (black bars, left Y-axis). These 50 HIV-1 subtype A sera showed the most frequent overall neutralization of the 2 subtype CRF02_AG viruses (total of 85% positives), followed by a frequency of 72% against the 3 combined subtype A viruses. Positive response rates against subtypes B, C, and D were 57%, 42% and 22%, respectively. The data set for subtype D was small with only 50 values against one subtype D virus. We therefore assessed whether the frequency of subtype A serum neutralization of subtype A and CRF02_AG viruses was greater than that observed against non-A viruses, as might be expected if subtype plays a role in neutralization [48], [49]. Grouping together the five A and CRF02_AG viruses and grouping the five non-A (B, C, and D) viruses together, the number of positives within the 250 tests (50 sera×5 viruses) in each subtype grouping was scored. The differences between subtype A serum neutralization of A and CRF02_AG viruses (77% positive) versus neutralization of non-A (B, C, D) viruses (44% positive) was highly significant (p<0.001, Fisher's exact test). In addition, the geometric mean ID_50_ values for all 50 subtype A sera against the 1–3 viruses within each subtype were calculated. The 50 subtype A sera showed the greatest overall potency (about 2-fold higher geometric means) against subtypes A and CRF02_AG (Fig. 5, white bars). The error bars represent the 95% confidence intervals. Overall, the potencies of subtype A serum NAb responses were ranked in the following order: CRF02_AG>A>D>C>B, with corresponding geometric mean ID_50_s of 243>238>126>106>98, respectively, (Fig. 5, right hand Y-axis, white bars). These observations require further analyses as the numbers of viruses were small (5 each of A versus non-A pseudoviruses) and there was only one subtype D virus in the panel.

### Correlation between gp120 binding antibody titers and NAb titer and breadth in sera from HIV-1 subtype A infected Ugandans

**Figure 6:**
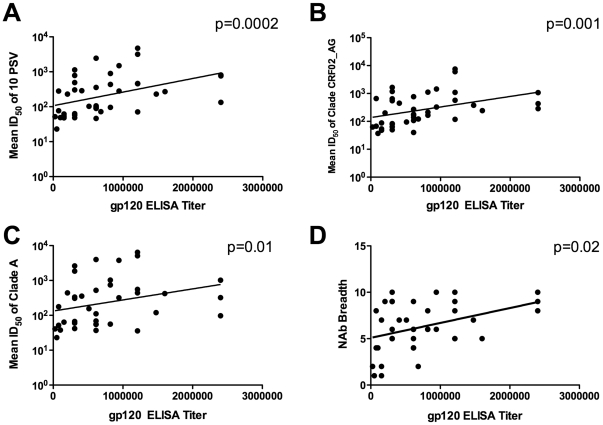
Binding antibodies against HIV-1 gp120 correlate with mean NAb Titer and breadth of neutralization. Subtype A gp120 binding antibody titers correlate with the mean NAb titers against all 10 pseudoviruses (A) and the mean ID_50_s of subtype CRF02_AG (B) and subtype A (C) pseudoviruses. A significant correlation was also seen between the gp120 binding antibody titers and neutralization breadth, as measured by the number of viruses neutralized (D).

IgG binding antibodies against HIV-1 subtype A gp120 were measured by ELISA using the 50 sera from HIV-1 subtype A infected Ugandans. The titers ranged from 25,600 to 24,000,000 with a mean of 714,971. Interestingly, a significant correlation was observed between gp120 IgG binding titer and mean NAb titer against all ten pseudoviruses (Spearman, p = 0.0002, rho = 0.54, Fig. 6A). A significant correlation was also found between gp120 binding and mean NAb titer against viruses from subtypes A (Spearman, p = 0.01, rho = 0.41) and CRF02_AG (Spearman, p = 0.001, rho = 0.5, Fig. 6C and B, respectively). In addition, a significant correlation could be seen between gp120 binding titer and the ID_50_ against pseudoviruses from HIV-1 subtype C (p<0.03) and against the individual pseudoviruses 92RW020.2 (subtype A), Du172.17 (subtype C) and PT271.11 (subtype CRF02_AG), all with p<0.03. The gp120-binding antibody titers also correlated with the breadth of neutralization, as measured by the number of viruses neutralized (out of 10) (Spearman, p = 0.02, rho = 0.36, Fig. 6D). Of note, gp120 binding antibody titers did not correlate with absolute B cell counts, CD4 counts or viral load (data not shown).

### ADCC in sera from subtype A infected Ugandans

In optimizing the ADCC assay, we found that the subtype A gp120 available for use in the ELISA assays provided no positive responses, presumably due to poor binding to the CEM.NK^r^ target cells. Thus, the ADCC assay was established using the subtype B gp120 used in previous studies by our laboratories [47], [50]. To establish a negative cut-off for the ADCC assay, ADCC activity was measured for the HIV-1 positive control sera and compared with activity in the negative controls. The mean % specific lysis for HIV+ controls was 37.7% (range 27.7–49.7%), as compared to 5.6% (range 3.1–8.3%) for the negative controls. Based on this, the mean ratio of the negative control after normalization was 0.15. Considering experimental error, we defined a negative response as one with a ratio of <0.2 and positives as a ratio of ≥0.2. Overall, 46/50 samples had demonstrable ADCC activity against targets coated with subtype B gp120. The mean ratio for ADCC specific lysis for samples that showed ADCC responses was 0.67 as compared to a ratio of 0.1 for the ADCC negative samples. However, ADCC responses did not correlate with absolute B cell counts (Spearman, p = 0.26), B cell percentage (Spearman, p = 0.12), absolute CD4+ T cell Counts (Spearman, p = 0.78) or viral load (Spearman, p = 0.16). In addition there was no relationship observed between ADCC and mean NAb titer (Spearman, p = 0.19) or gp120 binding antibodies (Spearman, p = 0.64) (data not shown).

## Discussion

Here we report significant peripheral blood B lymphopenia in HIV-1 subtype A infected Ugandans (Fig. 1), supporting several studies that have demonstrated B cell deficiencies and dysfunction in chronic HIV-1 infection [6], [7], [51], [52], [53], [54], [55], [56], [57], [58], [59], [60]. In addition, the profiles of gp120 binding and functional antibodies were assessed in sera from this cohort of Ugandan patients. Comparisons were made between B cell counts, viral load and CD4 numbers (Fig. 2) in order to determine the impact of the reduced peripheral B cell numbers on antibody functions and on disease stage in HIV-1 subtype A infection. High titers of NAbs [25] and potent ADCC-specific responses [33] have been demonstrated in chronic HIV infection, and a number of studies have investigated the prognostic role of these antibodies [23], [25], [29], [31], [32], [34], [35], [36]. The inverse correlation observed between CD4+ T cell count and viral load supports the expected relationship between these markers and is consistent with previous studies that demonstrate CD4 T cell decline as the hallmark of progressive HIV infection [1], [2]. Our findings suggest that this rural Ugandan population with chronic untreated subtype A HIV-1 is similar to other HIV-infected populations, where CD4+ T cell decline, increased viral load and subsequent immune dysfunction have been observed. The correlation between absolute B cell and CD4 T cell counts (Fig. 2a) also supports previous findings in chronically infected individuals [52]. Recently, it has also been shown that SIV infection in macaques results in a loss of both naïve and memory B cells during and after acute stages of infection [61].

Viral load did not show an inverse relationship with B cell counts, which might have been expected (Fig. 2). While B cells are not the target cells for virus replication, the profile of B cell phenotypes in peripheral blood is perturbed in HIV-1 infection [4]. Increased viral load results in B cell co-stimulatory dysfunction in chronic HIV infection [62], which can be measured early during primary infection [51]. HIV-1 has been shown to be capable of attaching to B cells via interactions between the CR2 complement receptor (CD21) and complement proteins bound to the virus. This interaction may provide stimulatory signals to B cells and, more importantly, may facilitate the transmission of HIV-1 to CD4+ T cells [4]. It has also recently been proposed that CD40L-bearing HIV-1 particles expressed by productively infected CD4+ T cells may play a role in the virus-induced humoral immune dysfunction by chronic activation of B cells through sustained CD40 signalling [10]. The lack of correlation of viral load and B cell number suggests that the virus is not specifically driving B cell depletion, but rather supports a model where CD4+ T cell decline diminishes the help that B cells require. It has been demonstrated that high levels of antigenic stimulation, as well as greater envelope genetic diversity early in infection, may generate broader NAb breadth, which is accompanied by a higher viral load set point [28].

In this study, no correlation was found between anti-gp120 binding antibodies and absolute B cell counts, viral load or CD4+ T cell counts, but there was a strong correlation of subtype A gp120 binding antibodies with Nab titer and breadth. These findings are in agreement with results from a longitudinal study characterizing the evolution of humoral responses in Thai patients infected with subtype CRF01_AE [26]. The correlation between neutralizing and binding antibody titers (Fig. 6B) as well as binding antibody titers and breadth (Fig. 6D) is important in the context of characterizing factors in HIV-1 pathogenesis and progression that could be indicators or correlates of protection for vaccine development. The impact of functional and binding antibody responses on disease progression has not been fully characterized in all subtypes. Since high titers of NAbs emerge later during infection, it is possible that the initial titers of binding antibodies may predict generation and evolution of NAbs. However, the kinetics involved in the evolution of subtype A binding and NAbs cannot be examined in this study, and will require longitudinal follow-up of subtype A infected patients, as is planned in an ongoing clinical protocol to assess acute infection in Uganda, Tanzania, and Kenya.

In assessing a direct relationship between peripheral B cell numbers and functional antibodies, an intriguing inverse correlation was observed between the mean NAb titer (against ten pseudoviruses) and the absolute B cell count. The weak inverse correlation found may be partially explained by the events of B cell activation and maturation that are most probably occurring after exposure to sufficient doses of viral antigen(s). In response to ligation of their cell-surface immunoglobulin, naïve B cells undergo clonal expansion and differentiation, developing either into memory cells or antibody secreting cells (ASC). IgG ASCs mainly traffic to the bone marrow or inflammatory sites, irrespective of their site of induction [63]. Thus, while these B cells are potentially secreting important functional antibodies, they may be trafficked out of the peripheral blood compartment, thus leading to decreased numbers of CD19+ B cells identified in peripheral blood, in the setting of antibody production against increasing epitopic exposure. As these B cells exit out of the peripheral circulation they may also become sequestered in secondary lymphoid tissue as infection progresses.

Of note, Beniguel et al. have demonstrated the potential for intact functional humoral responses, despite major B cell perturbations induced by HIV-1 and other tropical pathogens, in chronically infected, drug-naïve African subjects [64]. Thus, it is likely that functional antibodies against HIV-1 are detected (even at elevated levels) concomitant with a decline in the numbers of CD19+ peripheral B cells. Indeed, this is the first demonstration of an actual inverse relationship between B cells remaining in peripheral blood and HIV-1 NAb responses against subtype-matched and heterologous viruses. It is also important to note that in this study, only CD19+ B cells were enumerated and no additional phenotypic characterization of specific B cell sub-populations was performed. Future studies should aim to perform in depth characterization of the frequencies of specific populations of B cells to achieve a clearer understanding of their potential role(s) in functional responses.

In this study we demonstrate heterologous responses across different subtypes, consistent with previously reported findings [65], [66], as well as recent results from our laboratory demonstrating strong responses against subtype CRF02_AG viruses in Ugandan sera (Ouma et al., manuscript in preparation). A subset (approximately 10%, Fig. 4) of the subtype A serum samples in the present study demonstrated neutralization of all ten viruses representing five subtypes, and this is in agreement with the findings of neutralization breadth observed in other studies [67], [68], [69]. Blish et al. described the presence of cross-neutralizing antibodies in Kenyan plasma from subjects infected with subtypes A, C and D against viruses from multiple subtypes [70]. These broadly neutralizing antibodies may be a result of the targeting of conserved epitopes, alternatively, these samples might have been derived from later in chronic infection, allowing for the accumulation of a library of neutralizing antibodies representing the history of epitopes displayed by the infecting virus. This notion is also supported by findings in longitudinal studies in progression cohorts [71], [72], [73], although these studies involved subtype B infected long-term non-progressors and the use of T cell line-adapted (TCLA) strains or primary isolates.

Our findings also support some degree of subtype-specificity in the cross-clade neutralization observed. The subtype A sera showed significantly more frequent neutralization of, and higher geometric mean ID_50_s against, pseudoviruses with subtype A-containing envelopes (Figs. 4 and 5). While these data should be further substantiated with a larger virus panel (ie. only one subtype D virus was tested), the findings are important and suggestive regarding the influence of HIV-1 subtype in humoral immune responses. A growing number of studies have demonstrated that subtype can play a role in the specificity, frequency and perhaps magnitude of HIV-1 neutralization [48], [49], [74]. It has also been proposed that HIV-1 subtypes may vary in rates of disease progression [75], and this has been supported by data demonstrating that subtype D is associated with more rapid progression to AIDS [76], [77]. It will be important to determine the profiles of neutralizing antibody magnitude and breadth in subjects infected with subtype A, as this subtype is quite prevalent, particularly in Africa, and subtype A is a component of the two circulating recombinant forms (CRF01_AE and CRF02_AG) included amongst the major subtypes in the pandemic [41].

The failure of ADCC responses to correlate with B cell counts, viral load, CD4+ T cell counts and functional antibodies is a bit perplexing in light of findings in vaccinated and infected non-human primates where correlations between ADCC and viral load have been observed [37], [78], [79]. However, the ADCC assay employed offers low sensitivity and is a semi-quantitative assay [80]. Secondly, a number of epitopes within both gp120 and gp41 have been recognized to mediate ADCC activity [35], [81], [82]. In this study, only recombinant gp120 (and not gp41) was used to assess ADCC responses. Most importantly, because the subtype A gp120 did not permit the measurement of ADCC, the assay was performed using a subtype B gp120, which may entirely explain the lack of correlations with ADCC observed using subtype A infected patient sera. Finally, in vivo, ADCC involves components of both the innate and humoral immune systems and here we examined only the antibody function, and not cellular effector function, in these subtype A infected patients. We have previously shown alterations to the NK cell compartment, the primary cells that mediate ADCC, in HIV-1 infected Ugandans [83], however, further work will be required to assess the influence of the breadth and magnitude of ADCC on disease progression in different HIV-1 subtypes.

Here we have demonstrated for the first time, what appears to be an inverse relationship between peripheral blood B cell numbers and the magnitude and frequency of functional NAbs in subtype A infected subjects. We further demonstrate preferential neutralization of subtype A and CRF02_AG viruses by subtype A sera from Uganda. Studies of this nature may yield important insights for defining the relationship between functional antibodies, B cell populations and other prognostic markers in regions where multiple non-B HIV-1 subtypes are prevalent. Future studies in well-controlled acute infection cohorts will provide information that will augment these findings and better inform HIV vaccine development.
